# Evaluating the association of transferring governance of correctional health care services with overdose and all-cause mortality: a retrospective cohort study in British Columbia, Canada

**DOI:** 10.1186/s40352-025-00389-7

**Published:** 2025-11-22

**Authors:** Lisa McQuarrie, Dibbya Pravas Dasgupta, Tonia Nicholls, Ruth Elwood Martin, Katherine E McLeod, Stuart Kinner, Leigh Greiner, Maureen Olley, Kate Roth, Heather Palis, Ashok Krishnamoorthy, Amanda Slaunwhite

**Affiliations:** 1 School of Population and Public Health, University of British Columbia, Vancouver, BC Canada; 2 Canadian Collaboration for Prison Health and Education, School of Population and Public Health, University of British Columbia, Vancouver, BC Canada; 3 Department of Psychiatry, University of British Columbia, Vancouver, BC Canada; 4 BC Mental Health and Substance Use Services , Provincial Health Services Authority, Vancouver, BC Canada; 5 Department of Family Medicine, Faculty of Health Sciences, McMaster University, Hamilton, Ontario, Canada; 6 Justice Health Group, Faculty of Health Sciences, Curtin University, Perth, WA, Australia; 7 Justice Health Group, Murdoch Children’s Research Institute, Melbourne, VIC, Australia; 8 BC Corrections, Ministry of Public Safety and the Solicitor General, Province of British Columbia, Victoria, BC Canada; 9 BC Centre for Disease Control, Vancouver, BC Canada

**Keywords:** Correctional health services, Prison health, Health care governance, Mortality, Overdose death, Incarceration, Administrative health data, Difference-in-differences

## Abstract

**Background:**

In many jurisdictions world-wide, the government agency that manages prisons also provides prison health care services. However, the World Health Organization (WHO) and United Nations (UN) have recommended that health ministries provide prison health care. In Canada, the province of British Columbia (BC) transferred responsibility for correctional health services to the health ministry in accordance with this guidance. The objective of this study was to estimate the association between the transfer in BC and all-cause and overdose mortality within 1 year of release from prison.

**Methods:**

We used a retrospective cohort study design employing the difference-in-differences (DiD) method to compare mortality among formerly-incarcerated people in the pre- and post-transfer periods against a matched community control group to control for province-wide trends in mortality. The data source was a longitudinal linkage of administrative databases. The DiD effect was estimated with survival time-to-event models.

**Results:**

In the formerly-incarcerated group (*N* = 6912), all-cause (3.7% vs 2.6%) and overdose (2.7% vs 1.7%) mortality in the first-year post-release decreased from the pre-transfer period to the post-transfer period, while mortality risk changed little in the community control group (*N* = 6881) during this time period (all-cause: 0.7% vs 0.9%; overdose: 0.4% vs 0.4%). The transfer was associated with statistically significant reductions in the hazards of all-cause mortality (DiD HR: 0.52, 95% CI: [0.32, 0.83]) and overdose mortality (DiD HR: 0.51, 95% CI: [0.26, 0.99]) in the first-year post-release.

**Conclusions:**

This study provides empirical evidence in support of WHO and UN guidance and indicates that the delivery of correctional health services by community health authorities may reduce deaths, particularly overdose deaths, among people released from correctional centres.

**Supplementary Information:**

The online version contains supplementary material available at https://doi.org/10.1186/s40352-025-00389-7.

## Introduction

In 2022, there were approximately 11.5 million people incarcerated globally, an increase of 5.5% from a decade prior (United Nations Office on Drugs & Crime, 2024) and approximately 36 000 people incarcerated in Canadian correctional centres each day (Government of Canada, n.d.). People who experience incarceration in Canada have complex and challenging health care needs (Canada et al., 2022; Kouyoumdjian & Orkin, 2020) with high rates of mental illness (Butler et al., 2022; Hensel et al., 2020), substance use disorder (Butler et al., 2022; Gan et al., 2021), communicable diseases (Stewart et al., 2015), and non-communicable health conditions (e.g. cardiovascular diseases, respiratory diseases, and diabetes) (Kouyoumdjian et al., 2016). Premature death among people with a history of incarceration is frequent and commonly caused by overdose, cardiovascular disease, cancer, suicide, and interpersonal violence (Borschmann et al., 2024). People with a history of incarceration have a high risk of fatal overdose, particularly in the first two weeks following release from prison (Gan et al., 2021; Keen et al., 2021).

In the majority of European and North American countries, correctional health care services are provided by the government agency that manages the correctional facility (McLeod et al., 2020; Piper et al., 2019; Pont et al., 2018); this is the case in 11 of 14 federal, provincial, and territorial correctional systems in Canada (McLeod et al., 2020) and 64% (30 of 47) of Council of Europe member states (Van Hout et al., 2024). The World Health Organization and United Nations have recommended that the health care organisation that provides community health care should also provide prison health care (United Nations Office on Drugs and Crime & World Health Organization Regional Office for Europe, 2013) in alignment with the Nelson Mandela Rules (rule 24) (UN General Assembly, 2016), which state that care in prisons should meet the same standards as those in the community, and be organized in close relationship with community-based health care services. This recommendation was based on ethical and legal reasoning, not empirical research.

In 2017, responsibility for health care delivery in the ten provincial correctional centres in BC was transferred from the ministry responsible for corrections, which had contracted health care services to a private for-profit provider, to the Provincial Health Services Authority (PHSA), a public health care organisation that delivers services on behalf of the Ministry of Health (McLeod et al., 2020; Ministry of Public Safety & Solicitor General, 2017). As part of the governance transfer, the PHSA implemented a number of system-level changes, including standardised, patient-focused care policies and training; new nursing positions specific to substance use and discharge planning; revised management structure; and upgraded health care facilities. Additionally, the budget for correctional health services increased from $22.4 million pre-transfer to $35 million post-transfer, although this was due in part to the opening of a new correctional centre. The PHSA introduced a focus on continuity of care for people moving from community to correctional centres and vice versa. The governance change we deem ‘the transfer’ encapsulates all facets of the care transformation that occurred when the PHSA accepted responsibility of correctional health services.

In Canada, people on remand or sentenced to less than two years are placed in provincial correctional centres, and those with longer sentences are placed in federal correctional centres. Provincially-incarcerated people tend to have short stays and frequent transitions to/from the community or other centres. This creates a difficult environment for health care staff to provide quality and continuous health care within, in addition to the security restrictions and high health needs of the patients.

There is very limited research on the impact of changes in prison health governance on health outcomes (McLeod et al., 2020; Van Hout et al., 2024). Two previous qualitative studies examined the transfer of health care services in BC from the perspective of corrections (McLeod et al., 2024) and health care leadership (McLeod et al., 2023). The objective of this quantitative study was to evaluate the association between the transfer in BC and all-cause and overdose mortality within one year of release from incarceration (primary analysis) and any time after release (secondary analysis).

## Methods

### Study design and data source

We conducted a retrospective cohort study using the difference-in-differences (DiD) method. The change in the incarcerated group between the pre-transfer period (January 1, 2015 to September 30, 2017) and post-transfer period (April 1, 2018 to December 31, 2020) was compared to the change in the community control group over the same time periods. The intervening six months between the pre- and post-transfer periods was considered the ‘during transfer’ period and were excluded so that the transfer fully completed before the post-transfer period began. The timeline was identified based on conversations with correctional health leadership. Because the transfer affected all provincial correctional centres in BC and we could not access data from other correctional systems, the control group was selected from the non-incarcerated community. The control group was used to account for province-wide changes in mortality over the study period, during which overdose deaths were fluctuating (BCCDC, 2025) and the COVID-19 pandemic began (March 2020).

The data source for this study was the BC Provincial Overdose Cohort (BC-ODC), a linked longitudinal administrative database (MacDougall et al., 2019). The BC-ODC has a reference cohort containing a 20% random sample of British Columbia residents who were alive and registered for BC’s public health plan at some time between 2015 and 2020. The BC-ODC includes physician billing records, hospital admissions, emergency department visits, pharmacy dispensations, provincial incarceration records, provincial social assistance records, and provincial death records. Longitudinal data on individuals included in the BC-ODC were available from January 1, 2010 to December 31, 2020. Additional information on the BC-ODC is available elsewhere (MacDougall et al., 2019).

This study was approved by the University of British Columbia Behaviour Research Ethics Board (approval ID H23-02009). The study relied on the secondary use of deidentified data, therefore, participant consent was not required. The reporting of this study follows the Strengthening the Reporting of Observational Studies in Epidemiology (STROBE) guidelines (von Elm et al., 2007). Data were prepared using SAS version 9.2 (SAS®9.2, 2008) and analyses were conducted in R Statistical Software version v4.1.0 (R Core Team, 2021).

### Eligibility criteria

Individuals in the BC-ODC Reference Cohort were assigned an incarceration status (incarcerated or community) based on whether they had an eligible incarceration record between January 1, 2015 and December 31, 2020. To be eligible, incarceration records had to be longer than one day with known admission and release dates. Incarceration records from intermittent sentences, which are served only on weekends, and records with data entry errors were excluded (see Additional File 1 Section B for details). People with at least one eligible incarceration record were included in the incarcerated group if they were older than age 18 at admission, had sex recorded, and had not died during incarceration. For individuals with multiple eligible incarcerations, the last incarceration was selected because we theorized that the health care provided during the last incarceration would have a far greater impact on mortality post-release than earlier incarcerations (as a sensitivity analysis, the analysis was repeated using the first incarceration instead of the last). People in the incarcerated group who were released on or before September 30, 2017 were assigned to the pre-transfer period and people who were admitted on or after April 1, 2018 were assigned to the post-transfer period.

For the community control group, we evaluated eligibility yearly at January 1^st^ of each year between 2015 and 2020. We required controls to be at least age 18 and alive at January 1^st^ with sex recorded. Each community control contributed a record to the pool of potential controls for each year they met the eligibility criteria.

Propensity score matching was applied to the sample of eligible incarcerated and community individuals to obtain the final sample (see later section on matching for details). Controls were assigned to the same period (pre- or post-transfer) as their matched incarcerated person. After matching, individuals whose incarceration overlapped with the during transfer period were excluded along with their matched controls. For the primary analysis of mortality within 1 year of release, we excluded all incarcerated individuals whose last incarceration was after December 31, 2019 along with their matched controls, so that a full year of follow-up before the data cut-off was available for all included individuals (we also repeated the primary analysis with this restriction removed). No restrictions on the length of follow-up were applied in the secondary analysis of mortality any time after release.

### Outcome measure

Mortality was modelled as a time-to-event outcome as the number of days from the index date until death, or the end of the follow-up period if censored. The index date was the date of release from their last incarceration for incarcerated individuals or the release date of their matched incarcerated individual for community controls. If no death occurred within the follow-up period, the observation was censored. For the primary analysis of death within one year of the index date, the follow-up period was one year (365 days) from the index date. For the secondary analysis of death any time after the index date, the follow-up period was until the data cut-off date (December 31, 2020). In the analysis of overdose death, non-overdose death was treated as a competing event. The outcome was the time until death and observations were censored at the death date if the competing event occurred. Overdose was the most common cause of death among the study population, and no other causes of death occurred frequently enough to examine specifically.

### Other measures

The following covariates were included in the analysis and propensity score models: sex (male, female), age (< 30, 30–39, 40–49, 50–59, ≥ 60), calendar year, rural status (metro, urban, rural/remote, unknown), income assistance (no, yes), no fixed address (no, yes), opioid use disorder (OUD) (no, yes), Elixhauser comorbidity index (Elixhauser et al., 1998) (0, 1, ≥ 2), and mental illness (no, yes). The Elixhauser comorbidity index counts the number of comorbidities present across multiple categories; we removed the mental health categories from the index to capture physical comorbidities only. The Elixhauser comorbidity index and mental illness variables were calculated using all prior years of data. The income assistance, no fixed address, and OUD variables were calculated using the past year of data and the rural status variable was calculated using location in the index year. Additional details are available in the Additional File 1 Section C.

### Matching

Propensity score matching was used to select the control group from the pool of eligible potential controls. The propensity score model was a logistic regression, with a binary outcome for group (incarcerated vs control) and the variables listed in the previous section as covariates. The nearest neighbour method was used with a variable ratio of two controls to one incarcerated person. Exact matches were required on sex and year. The covariate balance between the two groups was assessed using standardised mean differences (SMDs) and SMDs of less than 0.1 were targeted (Austin, 2009).

Because eligibility criteria were evaluated on January 1^st^ before matching, a small number of matched controls had died between January 1^st^ and their index date (which was assigned after matching). These controls were excluded from the analysis (Suissa, 2007).

### Statistical analysis

Descriptive analyses included a Kaplan–Meier (KM) plot for all-cause mortality and cumulative incidence function (CIF) plots for overdose and non-overdose deaths (Koller et al., 2012). The 1-year mortality risk was calculated by group and period as the number of deaths within 1 year of the index date divided by the number of people in the group and period.

A Cox proportional-hazards (Cox PH) model was used to estimate the association between the transfer and all-cause mortality. A cause-specific hazard model for competing events was applied to analyse overdose mortality as the main event and non-overdose mortality as the competing event. The cause-specific hazard model is appropriate for investigating the relationship between exposure and time-to-event outcome when there are competing events (Koller et al., 2012). A separate model was estimated for each cause of death (overdose and non-overdose) and results for both causes are presented.

The DiD estimate is the coefficient of an interaction term between period and group from the model. The coefficient, after exponentiation, is the hazard ratio comparing the post-transfer to pre-transfer period in the incarcerated group, divided by the hazard ratio comparing the post-transfer to pre-transfer period in the control group. It can be interpreted as the excess change from pre-transfer to post-transfer in the incarcerated group compared to the change in the community group.

1 $$\begin{aligned} {\mathrm{D}}{\mathrm{i}}{\mathrm{D}} \, {\mathrm{e}}{\mathrm{f}}{\mathrm{f}}{\mathrm{e}}{\mathrm{c}}{\mathrm{t}} \, &\mathrm{=} \, \frac{\text{hazard incarcerated, pre-transfer}}{\text{hazard incarcerate}\text{d, post-transfer}}\\&\div \frac{\text{hazard community, pre-transfer}}{\text{hazard community, post-transfer}} \end{aligned}$$

DiD analysis relies on the parallel trends assumption (Angrist & Pischke, 2008), which we assessed by comparing pre-transfer trends. No evidence of non-parallel trends was found. Because variable ratio matching was used, matched controls were weighted with the reciprocal of the number of controls within the matched set (i.e. either 1 or 0.5) and incarcerated individuals had a weight of 1 (Stuart, 2010). Cluster robust standard errors were used to account for community controls that were matched in multiple years. The proportional hazard assumption was assessed using log–log survival curves and Schoenfeld residual plots, and was determined to be reasonable. The DiD coefficient was considered significant if the confidence interval did not include 1.

## Results

**Fig. 1:**
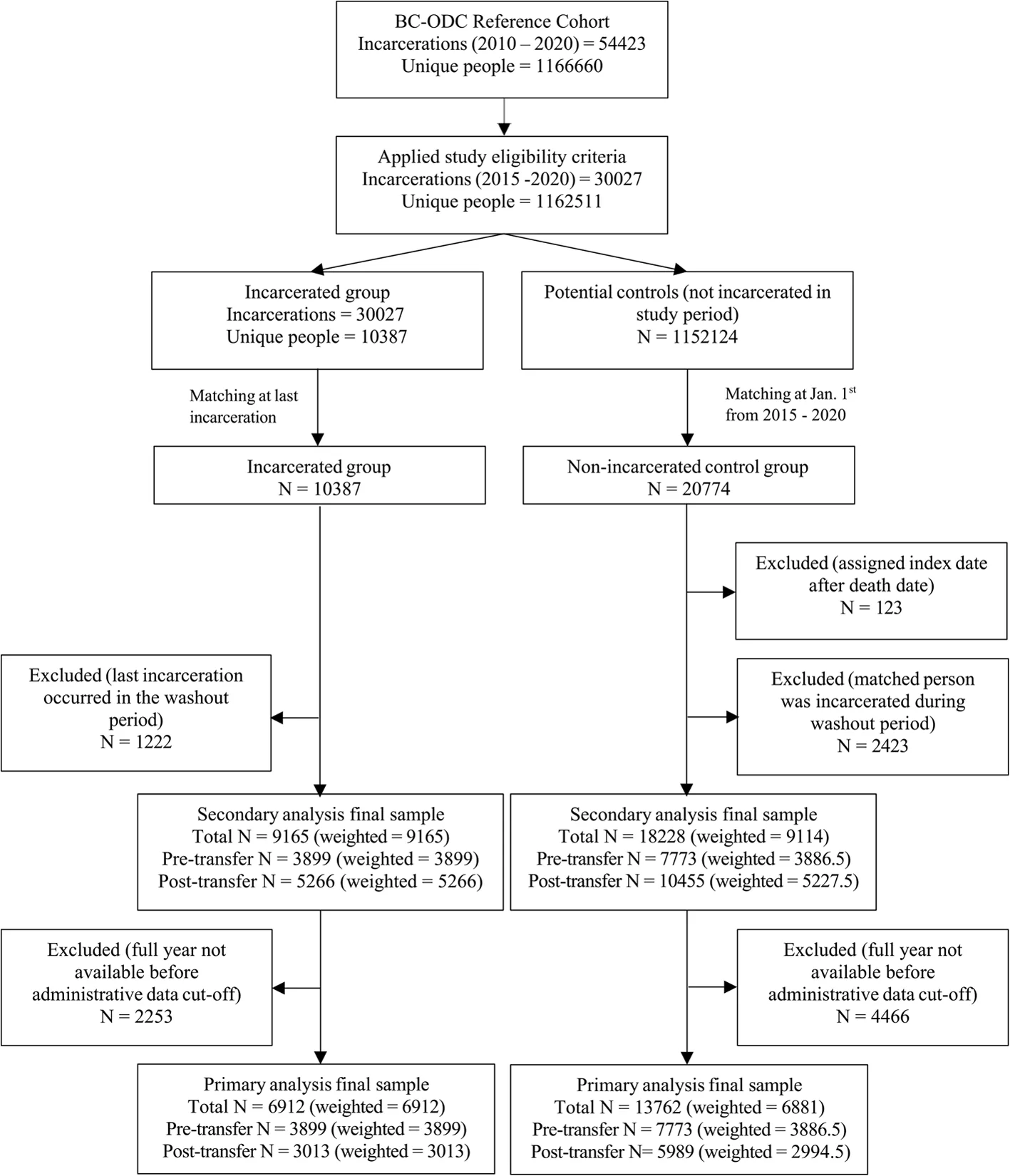
Flowchart illustrating the sample selection process

The BC-ODC Reference Cohort contained 1 166 660 unique people and 54 423 total incarcerations between 2010 and 2020. We filtered to 1 152 124 unique potential controls and 10 387 unique people who had been incarcerated during the study time frame. All incarcerated individuals were matched to a total of 20 774 community controls. After excluding observations that occurred during the washout period and controls who died before their assigned index date, the final matched community group contained 13 762 (weighted: 6 881) people for the primary analysis and 18 228 (weighted: 9 114) people for the secondary analysis. The final incarcerated group contained 6 912 people for the primary analysis and 9 165 people for the secondary analysis. Full details on the sample selection process are shown in Fig. 1.

The incarcerated group was majority male (pre-transfer: 85.4%; post-transfer: 85.6%) and young (median age pre-transfer: 36; post-transfer: 35) (Table 1). A large and increasing proportion of the incarcerated group suffered from mental illness (pre-transfer: 34.9%; post-transfer: 44.0%) and/or opioid use disorder (pre-transfer: 14.8%; post-transfer: 28.4%). Similarly, more people in the incarcerated group received income assistance (pre-transfer: 23.1%; post-transfer: 29.7%) and/or had no fixed address (pre-transfer: 10.8%; 20.4%) in the post-transfer period compared to the pre-transfer period. The matching was successful in creating a community control group with similar characteristics. The changes over time observed in the incarcerated group were accounted for by the matched control group, which exhibited similar increases from pre-transfer to post-transfer in the prevalence of mental illness, opioid use disorder, income assistance, and no fixed address.

**Table 1:** Description of the sample for the primary analysis

	Incarcerated		Community	
	Pre-transfer (*N* = 3899.0)	Post-transfer (*N* = 3013.0)	Pre-transfer (*N* = 3886.5)	Post-transfer (*N* = 2994.5)
Male sex (%)	3328.0 (85.4)	2580.0 (85.6)	3317.0 (85.3)	2565.0 (85.7)
Age (Median [IQR])	36 [28, 45]	35 [28, 45]	36 [28, 46]	37 [28, 47]
Age Category (%)				
< 30	1155.0 (29.6)	921.0 (30.6)	1148.0 (29.5)	843.5 (28.2)
30–39	1206.0 (30.9)	961.0 (31.9)	1164.5 (30.0)	863.0 (28.8)
40–49	927.0 (23.8)	661.0 (21.9)	902.0 (23.2)	661.0 (22.1)
50–59	447.0 (11.5)	358.0 (11.9)	470.5 (12.1)	446.5 (14.9)
≥ 60	164.0 (4.2)	112.0 (3.7)	201.5 (5.2)	180.5 (6.0)
Rural Status (%)				
Metro	993.0 (25.5)	690.0 (22.9)	1069.0 (27.5)	877.5 (29.3)
Urban	661.0 (17.0)	569.0 (18.9)	803.5 (20.7)	704.5 (23.5)
Rural/Remote	253.0 (6.5)	197.0 (6.5)	328.0 (8.4)	275.0 (9.2)
Unknown	1992.0 (51.1)	1557.0 (51.7)	1686.0 (43.4)	1137.5 (38.0)
Income Assistance (%)	899.0 (23.1)	895.0 (29.7)	952.0 (24.5)	1008.0 (33.7)
No Fixed Address (%)	422.0 (10.8)	616.0 (20.4)	375.5 (9.7)	508.5 (17.0)
Opioid Use Disorder (%)	577.0 (14.8)	856.0 (28.4)	645.0 (16.6)	813.0 (27.1)
Elixhauser Comorbidity w/o Mental Health (%)				
None	3542.0 (90.8)	2643.0 (87.7)	3533.5 (90.9)	2640.0 (88.2)
1	159.0 (4.1)	164.0 (5.4)	178.0 (4.6)	166.0 (5.5)
≥ 2	198.0 (5.1)	206.0 (6.8)	175.0 (4.5)	188.5 (6.3)
Mental illness (%)	1361.0 (34.9)	1325.0 (44.0)	1467.0 (37.7)	1459.0 (48.7)
Number of Prior Incarcerations (Median [IQR])	1 [0, 3]	2 [0, 5]	-	-
Length of Incarceration (days) (Median [IQR])	10 [4, 44]	12 [4, 44]	-	-

Table describes the characteristics of the matched sample included in the primary analysis of mortality within 1 year of the index date. Matching weights are applied. Decimal frequencies are due to the matching weights

*IQR* Interquartile Range

**Fig. 2:**
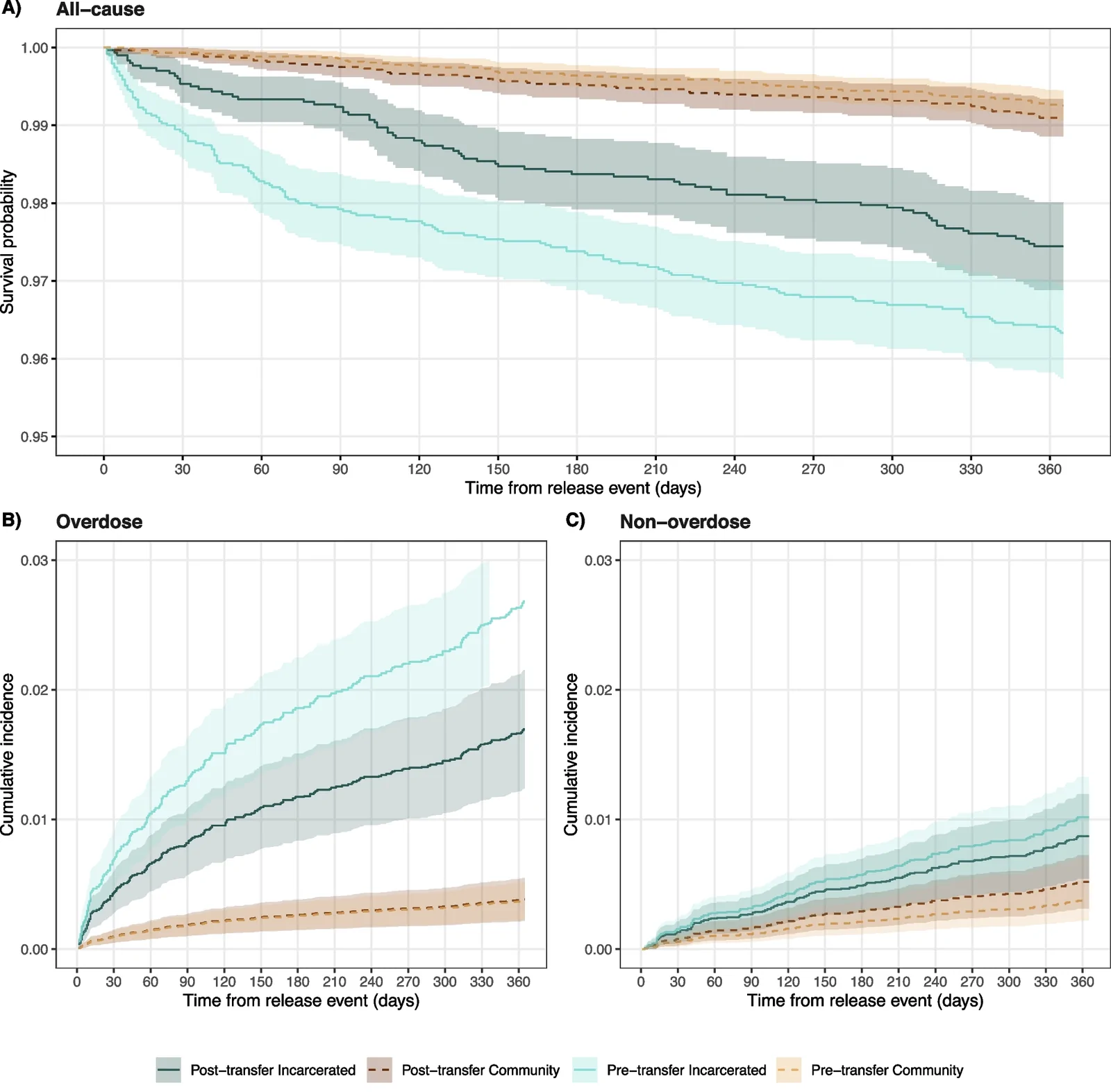
Survival plots for all-cause, overdose, and non-overdose mortality within 1 year of index date (weighted). **A** Kaplan–Meier plot for all-cause mortality within 1 year of index date. **B** Cumulative incidence plot for overdose mortality within 1 year of index date. **C** Cumulative incidence plot for non-overdose mortality within 1 year of index date. Matching weights were applied in all plots. A table with the number at risk and number of events over time is available in the Additional File 1 Section A (Table S2)

The all-cause mortality risk within 1 year of release for the incarcerated group declined from 3.7% before the transfer to 2.6% after the transfer (Table 2). The Kaplan–Meier plot (Fig. 2A) illustrates that individuals incarcerated after the transfer were less likely to die during their first-year post-release compared to those incarcerated before the transfer.

**Table 2:** Deaths occurring within 1 year of index date by group and period (weighted)

	Incarcerated		Community	
	Pre-transfer (*N* = 3899)	Post-transfer (*N* = 3013)	Pre-transfer (*N* = 3886.5)	Post-transfer (N = 2994.5)
All-cause	143.0 (3.7%)	77.0 (2.6%)	29.0 (0.7%)	27.0 (0.9%)
Overdose	104.0 (2.7%)	51.0 (1.7%)	14.5 (0.4%)	11.5 (0.4%)
Non-overdose	39.0 (1.0%)	26.0 (0.9%)	14.5 (0.4%)	15.5 (0.5%)

Percentages calculated as the number of deaths out of the number of people in the group and period. Matching weights are applied. Decimal frequencies are due to the matching weights

Overdose was the most common cause of death in the incarcerated group. The reduction in all-cause mortality can be partitioned into large reductions in overdose mortality and minimal reductions in non-overdose mortality. In the incarcerated group, overdose deaths within the first year of release declined from 2.7% pre-transfer to 1.7% post-transfer (Table 2). Figure 2B shows that the cumulative incidence of overdose death among formerly-incarcerated individuals was higher before the transfer than after the transfer. In the community group, there were minimal changes in mortality risk over the time period.

The DiD estimate for all-cause mortality within 1 year was 0.52 (95% CI: [0.32, 0.83]), indicating a 48% reduction in the hazard of mortality associated with the transfer (Table 3). We also identified a 49% reduction in the hazard of overdose mortality within 1 year associated with the transfer (DiD: 0.51, 95% CI: [0.26, 0.99]). A smaller and not statistically significant 34% reduction in the hazard of non-overdose mortality within 1 year was identified (DiD: 0.66, 95% CI: [0.32, 1.33]).

**Table 3:** Difference-in-differences estimates for primary and secondary analyses of all-cause, overdose, and non-overdose mortality

		**Cox PH**	**Cause-specific Hazard**	
		**All-cause**	**Overdose**	**Non-overdose**
		**DiD HR (95% CI)**	**DiD HR (95% CI)**	**DiD HR (95% CI)**
1 year post-release^a^	Unadjusted	0.57 (0.36, 0.91)	0.61 (0.32, 1.18)	0.62 (0.30, 1.26)
	Adjusted	0.52 (0.32, 0.83)	0.51 (0.26, 0.99)	0.66 (0.32, 1.33)
Any time post-release^b^	Unadjusted	0.86 (0.64, 1.17)	0.85 (0.56, 1.30)	0.89 (0.57, 1.38)
	Adjusted	0.93 (0.68, 1.25)	0.80 (0.52, 1.23)	1.10 (0.71, 1.71)

Results from a Cox proportional-hazards (PH) model for all-cause mortality and cause-specific hazard models for overdose and non-overdose mortality. Estimates were calculated using matching weights and robust standard errors

Unadjusted: Unadjusted model without any covariates

Adjusted: Model adjusted for sex, age, year, rural status, income assistance, no fixed address, opioid use disorder, Elixhauser comorbidity index, and mental illness

DiD HR: Difference-in-differences hazard ratio, estimate by an interaction term of period times group

^a^Mortality within 1 year of index date

^b^Mortality any time after index date (before end of study period)

In the secondary analysis of mortality any time after the index date, the DiD estimates for all three outcomes were smaller in magnitude and insignificant compared to the primary analysis (Table 3; additional secondary analysis results in the Additional File 1, Section A Table S1, Figure S1, and Table S4). In sensitivity analyses, the primary analysis was repeated without the requirement for the index date to be at least 1 year before the end of the study period and the results were unchanged (Additional File 1, Section A Table S5). The primary analysis was also repeated with the first incarceration selected for people with multiple incarcerations in the study period, as opposed to the last. Because there were fewer events occurring within 1 year of release, confidence intervals were wider and included one. Compared to the primary analysis with the last incarceration selected, the point DiD estimates for all-cause, overdose, and non-overdose mortality were closer to one, similar in magnitude, and above one, respectively (Additional File 1 Section A Table S6).

## Discussion

We found that the transfer of correctional health services to a public health care organisation in BC was associated with a decrease in the hazard of death within 1 year of release. The association of the transfer with mortality within 1 year was stronger than its association with mortality any time after release, indicating a more immediate than long-term association with health. The findings of this study demonstrate that the integration of correctional health with community health may reduce the risk of mortality and improve the health of formerly-incarcerated people.

As part of the transfer, the PHSA introduced many new practices and policies, including: new nursing roles that involved specialty care, such as discharge planning, substance use, and mental health; upgraded equipment; telehealth services; standardised quality improvement policies; and a new clinical services care plan that encouraged patient-centred care (McLeod et al., 2023). The budget for correctional health services also increased from $22.4 million to $35 million, in part because a new correctional centre had begun operating. Our results do not identify which of these specific aspects of the transfer led to reductions in mortality. A qualitative study that interviewed correctional health leadership after the transfer reported that integrating with community health programs strengthened the continuity of care between custody and community (McLeod et al., 2024). The observed reductions in mortality were largely due to a reduction in overdose deaths, which possibly indicates the success of overdose prevention initiatives introduced by the PHSA, such as improving access to opioid agonist treatment (OAT) during incarceration and upon release.

When interpreting the results of this study, there are several limitations to consider. To address confounding, we matched on many important variables, however, several social determinants of health were unmeasured, including gender (only sex was observed), education, and race/ethnicity/indigeneity. We used medical billing records to define many covariates, which may result in the misclassification of individuals who had limited contact with health care providers. The confidence intervals of estimates for cause-specific mortality were wide due to the small number of deaths during the study period. This study specifically examined deaths in the community after incarceration and did not include in-custody deaths because the risk of death during incarceration is much lower than the risk after leaving custody and in-custody deaths were rare during the study time period. The results can be easily generalized to other Canadian provinces/territories, but generalizing to other jurisdictions, including Europe, where changes of prison health governance are actively occurring (Van Hout et al., 2024), and the Canadian federal system, requires consideration.

This study has several strengths. The access to longitudinal, administrative data allowed a person-level DiD analysis with a matched, community control group to account for province-wide trends in mortality during the study period. The administrative data had limited missing or erroneous records. Access to longitudinal data allowed us to compare long-term health outcomes with several years of data before and after the transfer. The matching created a comparable community control group based on observed covariates.

## Conclusions

The results of this study provide evidence on the important role that prison health governance may have in the health of people who have been incarcerated. The observed association between the transfer and reductions in the hazard of death indicate the potential for benefits when prison health services are integrated within the broader health care system and illustrate the important role of correctional health services in preventing overdose deaths. With the growing population of people incarcerated globally and widening health inequities, the findings of this study reinforce the need for other jurisdictions in Canada and beyond to carefully consider and evaluate the governance of prison health care services within their regions or communities.

## Supplementary Information

Additional file 1. Supplement containing additional results and details on the study methodology.

## Data Availability

Data is unavailable for public distribution, please contact the BC Centre for Disease Control for information on how to access the BC Provincial Overdose Cohort.

## Abbreviations

BC: British Columbia
BC-ODC: British Columbia Provincial Overdose Cohort
CIF: Cumulative incidence function
COVID-19: Coronavirus disease of 2019
Cox PH: Cox proportional-hazards
DiD: Difference-in-differences
HR: Hazard ratio
IQR: Interquartile range
KM: Kaplan-Meier
OUD: Opioid use disorder
PHSA: Provincial Health Services Authority
SMD: Standardised mean difference
STROBE: Strengthening the reporting of observational studies in epidemiology
UN: United Nations
WHO: World Health Organization
