# Correction: Evaluating the association of transferring governance of correctional health care services with overdose and all-cause mortality: a retrospective cohort study in British Columbia, Canada

**DOI:** 10.1186/s40352-026-00459-4

**Published:** 2026-09-25

**Authors:** Lisa McQuarrie, Dibbya Pravas Dasgupta, Tonia Nicholls, Ruth Elwood Martin, Katherine E. McLeod, Stuart Kinner, Leigh Greiner, Maureen Olley, Kate Roth, Heather Palis, Ashok Krishnamoorthy, Amanda Slaunwhite

**Affiliations:** 1 School of Population and Public Health, University of British Columbia, Vancouver, BC Canada; 2 Canadian Collaboration for Prison Health and Education, School of Population and Public Health, University of British Columbia, Vancouver, BC Canada; 3 Department of Psychiatry, University of British Columbia, Vancouver, BC Canada; 4 BC Mental Health and Substance Use Services, Provincial Health Services Authority, Vancouver, BC Canada; 5 Department of Family Medicine, Faculty of Health Sciences, McMaster University, Hamilton, ON Canada; 6 Justice Health Group, Faculty of Health Sciences, Curtin University, Perth, WA Australia; 7 Justice Health Group, Murdoch Children’s Research Institute, Melbourne, VIC Australia; 8 BC Corrections, Ministry of Public Safety and the Solicitor General, Province of British Columbia, Victoria, BC Canada; 9 BC Centre for Disease Control, Vancouver, BC Canada

**Correction: Health Justice 14, 3 (2026)**

**https://doi.org/10.1186/s40352-025-00389-7**

Following the publication of the original article (McQuarrie et al. 2026), the author’s name Dibbya Pravas Dasgupta was incorrectly written as Dibbya Dasgupta.

This has been corrected above and the original article (McQuarrie et al. 2026) has been updated.
