# Supplementary material for: Evaluating the association of transferring governance of correctional health care services with overdose and all-cause mortality: a retrospective cohort study in British Columbia, Canada
Source: Health Justice. 2025 Nov 22;14:3. doi: 10.1186/s40352-025-00389-7 (PMC12809955; doi:10.1186/s40352-025-00389-7)
Supplement: Supplementary file 1 — Additional file 1. Supplement containing additional results and details on the study methodology. [file 40352_2025_389_MOESM1_ESM.docx]

**Table of Contents**

[Section A: Additional Analyses 3](#_Toc205550983)

[**Table S1:** Description of the sample for the secondary analysis 3](#_Toc205550984)

[**Table S2:** Table of number at risk and number of events for mortality within 1 year of the index date, by cause, period, and group 4](#_Toc205550985)

[**Figure S1:** Survival plots for all-cause, overdose, and non-overdose mortality any time after index date (weighted) 5](#_Toc205550986)

[**Table S3:** Modelling results for all-cause, overdose, and non-overdose mortality within 1 year of index date 6](#_Toc205550987)

[**Table S4:** Modelling results for all-cause, overdose, and non-overdose mortality any time after the index date 7](#_Toc205550988)

[**Table S5:** Modelling results for all-cause, overdose, and non-overdose mortality within 1 year of index date without requirement for 1 year of follow-up before the data cut-off 8](#_Toc205550989)

[**Table S6:** Difference-in-differences estimates for all-cause, overdose, and non-overdose mortality within 1 year of index date, using the first of multiple incarceration records 9](#_Toc205550990)

[Section B: Eligibility Criteria 10](#_Toc205550991)

[Section C: Algorithms for Covariate Definitions 12](#_Toc205550992)

[**C.1 Opioid Use Disorder (OUD)** 12](#_Toc205550993)

[**C.2 Mental illness** 12](#_Toc205550994)

[**C.5 Rural Status** 13](#_Toc205550995)

[**C.6 Income Assistance** 13](#_Toc205550996)

[**C.7 No Fixed Address** 13](#_Toc205550997)

[Section D: Description of Datasets in BC Provincial Overdose Cohort 14](#_Toc205550998)

[References 15](#_Toc205550999)

## Section A: Additional Analyses

### **Table S1:** Description of the sample for the secondary analysis

|  | **Incarcerated** | | **Community** | |
| --- | --- | --- | --- | --- |
|  | **Pre-transfer**  **(N=3899)** | **Post-transfer**  **(N=5266)** | **Pre-transfer**  **(N=3886.5)** | **Post-transfer**  **(5227.5)** |
| Sample Size | 3899 | 5266 | 3886.5 | 5227.5 |
| **Sex** = M (%) | 3328.0 (85.4) | 4631.0 (87.9) | 3317.0 (85.3) | 4596.5 (87.9) |
| **Age** (Median [IQR]) | 36 [28, 45] | 35 [28, 43] | 36 [28, 46] | 37 [29, 48] |
| **Age Category** (%) |  |  |  |  |
| <30 | 1155.0 (29.6) | 1561.0 (29.6) | 1148.0 (29.5) | 1404.0 (26.9) |
| 30-39 | 1206.0 (30.9) | 1844.0 (35.0) | 1164.5 (30.0) | 1568.5 (30.0) |
| 40-49 | 927.0 (23.8) | 1126.0 (21.4) | 902.0 (23.2) | 1154.5 (22.1) |
| 50-59 | 447.0 (11.5) | 562.0 (10.7) | 470.5 (12.1) | 781.0 (14.9) |
| >60 | 164.0 (4.2) | 173.0 (3.3) | 201.5 (5.2) | 319.5 (6.1) |
| **Rural Status** (%) |  |  |  |  |
| Metro | 993.0 (25.5) | 1247.0 (23.7) | 1069.0 (27.5) | 1573.0 (30.1) |
| Urban | 661.0 (17.0) | 1004.0 (19.1) | 803.5 (20.7) | 1257.5 (24.1) |
| Rural/Remote | 253.0 (6.5) | 343.0 (6.5) | 328.0 (8.4) | 495.5 (9.5) |
| Unknown | 1992.0 (51.1) | 2672.0 (50.7) | 1686.0 (43.4) | 1901.5 (36.4) |
| **Income Assistance** = Yes (%) | 899.0 (23.1) | 1637.0 (31.1) | 952.0 (24.5) | 1860.0 (35.6) |
| **No Fixed Address** = Yes (%) | 422.0 (10.8) | 1190.0 (22.6) | 375.5 (9.7) | 915.5 (17.5) |
| **OUD Status** = Yes (%) | 577.0 (14.8) | 1636.0 (31.1) | 645.0 (16.6) | 1536.0 (29.4) |
| **Elixhauser Comorbidity w/o Mental Health** (%) |  |  |  |  |
| None | 3542.0 (90.8) | 4612.0 (87.6) | 3533.5 (90.9) | 4554.5 (87.1) |
| 1 | 159.0 (4.1) | 312.0 (5.9) | 178.0 (4.6) | 320.0 (6.1) |
| ≥2 | 198.0 (5.1) | 342.0 (6.5) | 175.0 (4.5) | 353.0 (6.8) |
| **Mental Illness** = Yes (%) | 1361.0 (34.9) | 2499.0 (47.5) | 1467.0 (37.7) | 2760.5 (52.8) |
| **Prior Incarceration** (Median [IQR]) | 1 [0, 3] | 2 [0, 6] | - | - |
| **Length of Incarceration (days)** (Median [IQR]) | 10 [4, 44] | 17 [5, 58] | - | - |

Table describes the characteristics of the matched sample included in the secondary analysis of mortality any time after the index date. Matching weights are applied. Decimal frequencies are due to the matching weights.

IQR = Interquartile Range

### **Table S2:** Table of number at risk and number of events for mortality within 1 year of the index date, by cause, period, and group

|  |  |  | **Time from release event (days)** | | | | | | | | | | | | |
| --- | --- | --- | --- | --- | --- | --- | --- | --- | --- | --- | --- | --- | --- | --- | --- |
|  |  |  | **0** | **30** | **60** | **90** | **120** | **150** | **180** | **210** | **240** | **270** | **300** | **330** | **365** |
| Number at risk | Incarcerated | Pre-transfer | 3899.0 | 3857.0 | 3832.0 | 3818.0 | 3812.0 | 3803.0 | 3797.0 | 3789.0 | 3781.0 | 3774.0 | 3770.0 | 3764.0 | 3756.0 |
|  |  | Post-transfer | 3013.0 | 2999.0 | 2993.0 | 2990.0 | 2977.0 | 2968.0 | 2964.0 | 2962.0 | 2956.0 | 2954.0 | 2951.0 | 2941.0 | 2936.0 |
|  | Community | Pre-transfer | 3886.5 | 3884.0 | 3882.0 | 3881.0 | 3877.5 | 3874.5 | 3872.5 | 3870.5 | 3869.0 | 3867.0 | 3864.5 | 3862.0 | 3857.5 |
|  |  | Post-transfer | 2994.5 | 2992.5 | 2989.5 | 2987.0 | 2984.5 | 2982.0 | 2980.5 | 2978.5 | 2976.5 | 2975.5 | 2974.5 | 2972.5 | 2967.5 |
| Number of events (all-cause) | Incarcerated | Pre-transfer | 0.0 | 42.0 | 67.0 | 81.0 | 87.0 | 96.0 | 102.0 | 110.0 | 118.0 | 125.0 | 129.0 | 135.0 | 143.0 |
|  |  | Post-transfer | 0.0 | 14.0 | 20.0 | 23.0 | 36.0 | 45.0 | 49.0 | 51.0 | 57.0 | 59.0 | 62.0 | 72.0 | 77.0 |
|  | Community | Pre-transfer | 0.0 | 2.5 | 4.5 | 5.5 | 9.0 | 12.0 | 14.0 | 16.0 | 17.5 | 19.5 | 22.0 | 24.5 | 29.0 |
|  |  | Post-transfer | 0.0 | 2.0 | 5.0 | 7.5 | 10.0 | 12.5 | 14.0 | 16.0 | 18.0 | 19.0 | 20.0 | 22.0 | 27.0 |
| Number of events (overdose) | Incarcerated | Pre-transfer | 0.0 | 34.0 | 52.0 | 65.0 | 68.0 | 73.0 | 77.0 | 82.0 | 87.0 | 91.0 | 94.0 | 99.0 | 104.0 |
|  |  | Post-transfer | 0.0 | 10.0 | 14.0 | 16.0 | 25.0 | 31.0 | 34.0 | 35.0 | 37.0 | 38.0 | 41.0 | 48.0 | 51.0 |
|  | Community | Pre-transfer | 0.0 | 1.0 | 2.5 | 3.5 | 5.5 | 7.5 | 9.0 | 10.0 | 10.5 | 11.0 | 11.5 | 12.5 | 14.5 |
|  |  | Post-transfer | 0.0 | 1.0 | 2.0 | 3.0 | 3.5 | 5.0 | 5.5 | 6.0 | 7.5 | 8.5 | 8.5 | 9.0 | 11.5 |
| Number of events (non-overdose) | Incarcerated | Pre-transfer | 0.0 | 8.0 | 15.0 | 16.0 | 19.0 | 23.0 | 25.0 | 28.0 | 31.0 | 34.0 | 35.0 | 36.0 | 39.0 |
|  |  | Post-transfer | 0.0 | 4.0 | 6.0 | 7.0 | 11.0 | 14.0 | 15.0 | 16.0 | 20.0 | 21.0 | 21.0 | 24.0 | 26.0 |
|  | Community | Pre-transfer | 0.0 | 1.5 | 2.0 | 2.0 | 3.5 | 4.5 | 5.0 | 6.0 | 7.0 | 8.5 | 10.5 | 12.0 | 14.5 |
|  |  | Post-transfer | 0.0 | 1.0 | 3.0 | 4.5 | 6.5 | 7.5 | 8.5 | 10.0 | 10.5 | 10.5 | 11.5 | 13.0 | 15.5 |

Number at risk indicates the number of people without an event and not censored at the time point. Number of events is the cumulative number of events up to the time point. Matching weights are applied. Decimal frequencies are due to the matching weights.

### **Figure S1:** Survival plots for all-cause, overdose, and non-overdose mortality any time after index date (weighted)


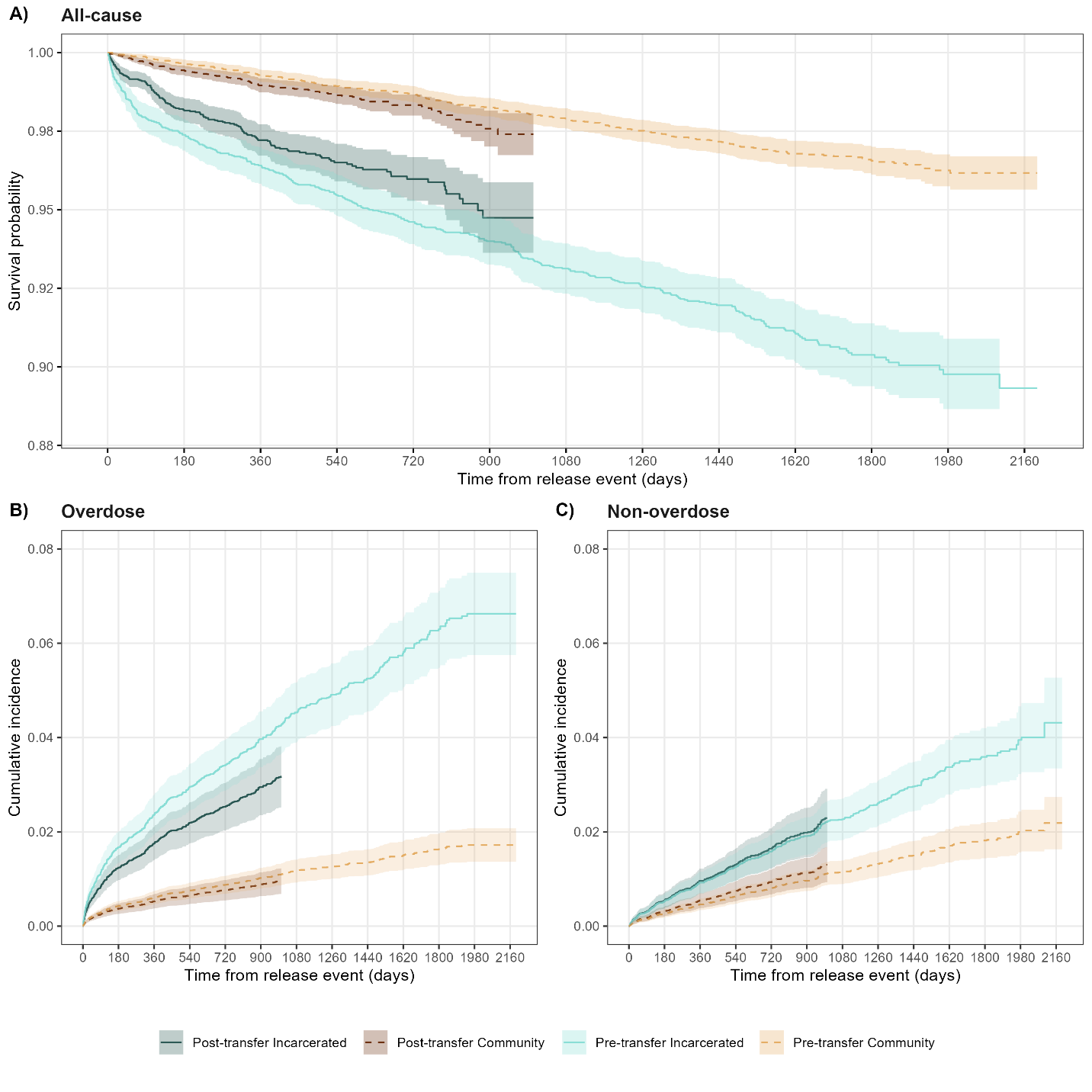


A) Kaplan-Meier plot for all-cause mortality any time after the index date. B) Cumulative incidence plot for overdose mortality any time after the index date. C) Cumulative incidence plot for non-overdose mortality any time after the index date. Matching weights are applied in all plots.

### **Table S3:** Modelling results for all-cause, overdose, and non-overdose mortality within 1 year of index date

|  | **Cox PH** | **Cause-specific Hazard** | |
| --- | --- | --- | --- |
|  | **All-cause** | **Overdose** | **Non-overdose** |
|  | **HR (95% CI)** | **HR (95% CI)** | **HR (95% CI)** |
| **Differences-in-Differences** |  |  |  |
| Period × Group | 0.52 (0.32, 0.83) | 0.51 (0.26, 0.99) | 0.66 (0.32, 1.33) |
| **Period** (Ref: Pre-transfer) |  |  |  |
| Post-transfer | 0.90 (0.61, 1.31) | 0.78 (0.44, 1.38) | 1.03 (0.62, 1.73) |
| **Group** (Ref: Community) |  |  |  |
| Incarcerated | 5.57 (4.11, 7.55) | 7.56 (4.97, 11.5) | 3.26 (2.04, 5.22) |
| **Sex** (Ref: Female) |  |  |  |
| Male | 1.69 (1.23, 2.32) | 2.08 (1.37, 3.15) | 1.12 (0.68, 1.86) |
| **Age Category** (Ref: <30) |  |  |  |
| 30-39 | 0.92 (0.67, 1.25) | 0.97 (0.68, 1.39) | 0.78 (0.43, 1.41) |
| 40-49 | 0.91 (0.65, 1.26) | 0.84 (0.56, 1.26) | 1.07 (0.60, 1.92) |
| 50-59 | 0.79 (0.53, 1.16) | 0.63 (0.37, 1.05) | 1.17 (0.63, 2.18) |
| >60 | 1.76 (1.14, 2.71) | 0.55 (0.21, 1.45) | 3.82 (2.12, 6.88) |
| **Rural Status** (Ref: Metro) |  |  |  |
| Urban | 1.45 (1.07, 1.95) | 1.28 (0.87, 1.91) | 1.67 (1.06, 2.63) |
| Rural/Remote | 0.89 (0.53, 1.47) | 0.67 (0.32, 1.40) | 1.23 (0.60, 2.53) |
| Unknown | 1.07 (0.80, 1.43) | 1.22 (0.86, 1.73) | 0.78 (0.47, 1.31) |
| **Income Assistance** (Ref: No) |  |  |  |
| Yes | 1.03 (0.75, 1.42) | 1.03 (0.68, 1.56) | 0.99 (0.61, 1.61) |
| **No Fixed Address** (Ref: No) |  |  |  |
| Yes | 2.08 (1.56, 2.78) | 1.78 (1.23, 2.58) | 2.59 (1.65, 4.07) |
| **OUD Status** (Ref: No) |  |  |  |
| Yes | 2.38 (1.74, 3.26) | 3.32 (2.18, 5.05) | 1.25 (0.77, 2.04) |
| **Elixhauser Comorbidity w/o Mental Health** (Ref: None) |  |  |  |
| 1 | 1.90 (1.31, 2.77) | 2.13 (1.38, 3.31) | 1.38 (0.69, 2.76) |
| ≥2 | 3.03 (2.24, 4.10) | 2.27 (1.47, 3.49) | 4.37 (2.81, 6.79) |
| **Mental illness** (Ref: No) |  |  |  |
| Yes | 1.84 (1.37, 2.47) | 2.03 (1.40, 2.95) | 1.48 (0.91, 2.39) |

Results from a Cox proportional-hazards (PH) model for all-cause mortality and cause-specific hazard models for overdose and non-overdose mortality within one year of index date. Estimates are from covariate-adjusted models with matching weights and robust standard errors.

HR: hazard ratio; CI: confidence interval; Ref: reference level

### **Table S4:** Modelling results for all-cause, overdose, and non-overdose mortality any time after the index date

|  | **Cox PH** | **Cause-specific Hazard** | | |
| --- | --- | --- | --- | --- |
|  | **All-cause** | | **Overdose** | **Non-overdose** |
|  | **HR (95% CI)** | | **HR (95% CI)** | **HR (95% CI)** |
| **Differences-in-Differences** |  | |  |  |
| Period × Group | 0.93 (0.68, 1.25) | | 0.80 (0.52, 1.23) | 1.10 (0.71, 1.71) |
| **Period** (Ref: Pre-transfer) |  | |  |  |
| Post-transfer | 0.70 (0.54, 0.89) | | 0.64 (0.44, 0.93) | 0.84 (0.60, 1.19) |
| **Group** (Ref: Community) |  | |  |  |
| Incarcerated | 3.39 (2.86, 4.02) | | 4.31 (3.37, 5.51) | 2.43 (1.89, 3.11) |
| **Sex** (Ref: Female) |  | |  |  |
| Male | 1.48 (1.20, 1.83) | | 1.72 (1.30, 2.29) | 1.16 (0.85, 1.59) |
| **Age Category** (Ref: <30) |  | |  |  |
| 30-39 | 0.97 (0.78, 1.20) | | 1.02 (0.79, 1.32) | 0.83 (0.55, 1.24) |
| 40-49 | 1.19 (0.95, 1.48) | | 1.10 (0.84, 1.43) | 1.42 (0.98, 2.08) |
| 50-59 | 1.34 (1.05, 1.72) | | 0.88 (0.62, 1.25) | 2.36 (1.63, 3.42) |
| >60 | 2.89 (2.22, 3.76) | | 0.86 (0.50, 1.50) | 6.33 (4.33, 9.26) |
| **Rural Status** (Ref: Metro) |  | |  |  |
| Urban | 1.07 (0.89, 1.29) | | 1.01 (0.78, 1.31) | 1.13 (0.86, 1.49) |
| Rural/Remote | 0.85 (0.64, 1.14) | | 0.66 (0.42, 1.03) | 1.04 (0.70, 1.54) |
| Unknown | 0.70 (0.57, 0.84) | | 0.82 (0.64, 1.04) | 0.52 (0.38, 0.72) |
| **Income Assistance** (Ref: No) |  | |  |  |
| Yes | 1.33 (1.09, 1.62) | | 1.33 (1.01, 1.75) | 1.28 (0.96, 1.70) |
| **No Fixed Address** (Ref: No) |  | |  |  |
| Yes | 1.75 (1.46, 2.11) | | 1.81 (1.42, 2.31) | 1.62 (1.22, 2.16) |
| **OUD Status** (Ref: No) |  | |  |  |
| Yes | 1.65 (1.36, 2.00) | | 1.99 (1.53, 2.58) | 1.21 (0.91, 1.61) |
| **Elixhauser Comorbidity w/o Mental Health** (Ref: None) |  | |  |  |
| 1 | 1.67 (1.30, 2.14) | | 1.60 (1.16, 2.21) | 1.78 (1.22, 2.60) |
| ≥2 | 2.71 (2.22, 3.32) | | 1.81 (1.32, 2.47) | 4.13 (3.15, 5.41) |
| **Mental illness** (Ref: No) |  | |  |  |
| Yes | 1.78 (1.48, 2.14) | | 2.14 (1.65, 2.76) | 1.35 (1.03, 1.77) |

Results from a Cox proportional-hazards (PH) model for all-cause mortality and cause-specific hazard models for overdose and non-overdose mortality any time after index date. Estimates are from covariate-adjusted models with matching weights and robust standard errors.

HR: hazard ratio; CI: confidence interval; Ref: reference level

### **Table S5:** Modelling results for all-cause, overdose, and non-overdose mortality within 1 year of index date without requirement for 1 year of follow-up before the data cut-off

|  | **Cox PH** | **Cause-specific Hazard** | |
| --- | --- | --- | --- |
|  | **All-cause** | **Overdose** | **Non-overdose** |
|  | **HR (95% CI)** | **HR (95% CI)** | **HR (95% CI)** |
| **Differences-in-Differences** |  |  |  |
| Period × Group | 0.53 (0.34, 0.81) | 0.50 (0.27, 0.92) | 0.67 (0.35, 1.27) |
| **Period** (Ref: Pre-transfer) |  |  |  |
| Post-transfer | 0.96 (0.67, 1.36) | 0.86 (0.51, 1.46) | 1.10 (0.69, 1.75) |
| **Group** (Ref: Non-incarcerated) |  |  |  |
| Incarcerated | 5.57 (4.11, 7.55) | 7.52 (4.95, 11.4) | 3.29 (2.05, 5.28) |
| **Sex** (Ref: Female) |  |  |  |
| Male | 1.68 (1.24, 2.26) | 1.96 (1.33, 2.89) | 1.22 (0.76, 1.97) |
| **Age Category** (Ref: <30) |  |  |  |
| 30-39 | 0.98 (0.73, 1.30) | 1.04 (0.74, 1.44) | 0.83 (0.47, 1.46) |
| 40-49 | 1.10 (0.82, 1.49) | 1.01 (0.71, 1.45) | 1.37 (0.80, 2.33) |
| 50-59 | 0.91 (0.64, 1.29) | 0.66 (0.41, 1.08) | 1.54 (0.88, 2.68) |
| >60 | 1.92 (1.29, 2.86) | 0.55 (0.23, 1.34) | 4.39 (2.49, 7.73) |
| **Rural Status** (Ref: Metro) |  |  |  |
| Urban | 1.34 (1.02, 1.76) | 1.14 (0.80, 1.63) | 1.72 (1.13, 2.61) |
| Rural/Remote | 0.94 (0.61, 1.45) | 0.58 (0.29, 1.17) | 1.56 (0.86, 2.83) |
| Unknown | 1.01 (0.77, 1.31) | 1.09 (0.79, 1.50) | 0.82 (0.51, 1.33) |
| **Income Assistance** (Ref: No) |  |  |  |
| Yes | 1.13 (0.85, 1.50) | 1.18 (0.81, 1.70) | 1.01 (0.65, 1.56) |
| **No Fixed Address** (Ref: No) |  |  |  |
| Yes | 2.09 (1.62, 2.71) | 1.90 (1.37, 2.64) | 2.35 (1.56, 3.54) |
| **OUD Status** (Ref: No) |  |  |  |
| Yes | 2.00 (1.51, 2.64) | 2.49 (1.71, 3.62) | 1.29 (0.84, 1.99) |
| **Elixhauser Comorbidity w/o Mental Health** (Ref: None) |  |  |  |
| 1 | 1.95 (1.41, 2.72) | 2.12 (1.43, 3.14) | 1.60 (0.90, 2.86) |
| ≥2 | 2.95 (2.24, 3.88) | 2.18 (1.47, 3.25) | 4.33 (2.92, 6.43) |
| **Mental illness** (Ref: No) |  |  |  |
| Yes | 1.82 (1.39, 2.38) | 1.96 (1.39, 2.75) | 1.55 (1.00, 2.39) |

Results from a Cox proportional-hazards (PH) model for all-cause mortality and cause-specific hazard models for overdose and non-overdose mortality within 1 year of the index date. The exclusion criteria requiring the index date to be at least 1 year before the data cut-off date was not applied. Estimates are from covariate-adjusted models with matching weights and robust standard errors.

Ref: reference level

### **Table S6:** Difference-in-differences estimates for all-cause, overdose, and non-overdose mortality within 1 year of index date, using the first of multiple incarceration records

|  | **Cox PH** | **Cause-specific Hazard** | |
| --- | --- | --- | --- |
|  | **All-cause**  **DiD HR (95% CI)** | **Overdose**  **DiD HR (95% CI)** | **Non-overdose**  **DiD HR (95% CI)** |
| 1-year post-release, adjusted | 0.79 (0.43, 1.44) | 0.54 (0.23, 1.27) | 1.35 (0.56, 3.22) |

Results from a Cox proportional-hazards (PH) model for all-cause mortality and cause-specific hazard models for overdose and non-overdose mortality within 1 year of the index date. Estimates were calculated using matching weights and robust standard errors. For people with multiple incarcerations, the first incarceration was selected. Model was adjusted for sex, age, year, rural status, income assistance, no fixed address, opioid use disorder, Elixhauser comorbidity index, and mental illness.

DiD HR: Difference-in-differences hazard ratio, calculated as the interaction between period and group.

## Section B: Eligibility Criteria

Person-level eligibility criteria for the incarcerated group:

- People with at least one eligible incarceration between January 1, 2015 and December 31, 2020 were included in the incarcerated group.
- People who died during incarceration were excluded.
- People without sex recorded were excluded.

Incarceration-level eligibility criteria:

- Incarcerations lasting more than one day were included.
- Incarcerations by adults (aged 18 or older at admission) were included.
- Incarcerations with missing release date were excluded.
- Incarcerations classified as intermittent sentences were excluded. People with intermittent sentences are usually incarcerated in a correctional centre during the weekend and released to the community during the week.
- Overlapping incarceration records on the same person were excluded. If there were multiple incarceration records from the same person where the dates indicated overlapping periods of incarceration (ie. the second admission date was before the first release date), all records were removed.

Individual-level eligibility criteria for the community control group:

- People without any incarceration records between January 1, 2015 and December 31, 2020 were eligible for the community group.
- People who were aged 18 or older at baseline were included.
- People who died before baseline were excluded.
- People without sex recorded were excluded.

Baseline was January 1^st^ and eligibility criteria were evaluated in each year between 2015 and 2020. Individuals were included in the pool of potential controls for each year they were eligible.

Exclusion criteria applied after matching:

- Matched controls who died before index date, which was the release date of their matched incarcerated person, were excluded.
- Incarcerated people whose last incarceration overlapped with the washout period from October 1, 2017 to March 31, 2018 were excluded along with their matched controls.
- For the primary analysis only, incarcerated people whose last incarceration occurred after December 31, 2019 were excluded along with their matched controls. This criterion was applied to exclude people who would not have the possibility of a full-year of follow-up before the data cut-off date on December 31, 2020.

## Section C: Algorithms for Covariate Definitions

### **C.1 Opioid Use Disorder (OUD)**

1. Physician billing records (Medical Services Plan [MSP]), hospitalization records (Discharge Abstract Database [DAD]), and pharmacy dispensation records (PharmaNet) were filtered to records with the ICD-9, ICD-10, and DIN/PIN codes shown in Table S6.^1^
2. All three data sets were filtered to records that occurred before the timepoint.
3. For MSP records, 2 records were required within the same 12-month period. If the later of the 2 records occurred within 1 year prior to the timepoint then the person was considered to have OUD. For DAD and PharmaNet records, if there exists one record within 1 year prior to the timepoint then the person was considered to have OUD.

**Table S7:** MSP and DAD codes for identifying OUD.

| **Data set** | **Codes** |
| --- | --- |
| MSP | 304.0, 304.7, 305.5 |
| DAD | F11 |
| PharmaNet | 999792, 999793, 66999990, 66999991, 66999992, 6999993, 66999997, 66999998, 6999999, 67000000, 67000001, 67000002, 67000003, 67000004, 67000005, 67000006, 67000007, 67000008, 67000009, 67000010, 67000011, 67000012, 67000013, 67000014, 67000015, 67000016, 67000017, 67000018, 67000019, 67000020, 2295695, 2295709, 2408090, 2408104, 2424851, 2424878, 2453908, 2453916, 2468085, 2468093, 2502313, 2502321,  2502348, 2502356, 2517175, 2517183, 22123346, 22123347, 2123348, 22123349, 2019930, 2019949, 2019957, 2019965, 2177749, 2177757 |

### **C.2 Mental illness**

For each of anxiety, depression, schizophrenia, bipolar, and stress disorder, if there were at least 2 physician billing records (MSP) containing the relevant ICD-9 codes shown in Table S7 within the same 12-month period or 1 hospitalization record (DAD) with the relevant ICD-10 code shown in Table S7 at any time prior to the relevant timepoint then the person was considered to have mental illness.^2^

**Table S8:** Definition of mental illness indicator.

| **Health condition** | **Database** | **Codes** |
| --- | --- | --- |
| Anxiety | MSP | 300 excluding 300.4 and 50B |
|  | DAD | F40, F41 |
| Depression | MSP | 300.4, 311, 50B |
|  | DAD | F32, F33, F34.1 |
| Schizophrenia | MSP | 295, 297, 298 |
|  | DAD | F20, F21, F22, F23, F24, F25, F28, F29 |
| Bipolar | MSP | 296 |
|  | DAD | F30, F31, F34, F38, F39 excluding F34.1 |
| Stress disorder | MSP | 308, 309 |
|  | DAD | F43 |

### **C.5 Rural Status**

The postal code for the relevant calendar year was linked to the local health service area in the BC Provincial Overdose Cohort Client Roster. Local health service areas are defined by the BC Ministry of Health and represent homogeneous regions by population and geographic location. If the local health service area was unknown for the relevant year then the last known value was carried forward; records with unknown local health service area in the current and all prior years were marked as unknown. Each local health area was assigned to metropolis, urban, rural, or remote based on the guidance document from the BC Ministry of Health.^3^ The rural and remote categories were then combined into one category.

### **C.6 Income Assistance**

If there was at least one pharmacy dispensation record (PharmaNet) within the 1 year prior to the timepoint with a plan class of ‘C’ or at least one record within the 1 year prior to the timepoint with income assistance indicated in the Social Development and Poverty Reduction (SDPR) data set, then the person was considered to have received income assistance.^1^ A plan class of ‘C’ in PharmaNet indicates that the prescription cost is fully covered by public health insurance because the recipient is receiving income assistance. The SDPR data set is provided by the Ministry of Social Development and Poverty Reduction and contains information about recipients of social assistance programs.

### **C.7 No Fixed Address**

If any of the following conditions were met then the person was considered to have no fixed address:^1^

1. at least one DAD record with the ICD-10 code Z59.0 or Z59.1
2. at least one MSP record with the ICD-9 code V60.0 or V60.1
3. at least 3 consecutive months marked as no fixed address in the SDPR data set

Records within 1 year prior to the timepoint were considered in the calculation.

## Section D: Description of Datasets in BC Provincial Overdose Cohort

**Ministry of Health Provincial Client Roster:**^1^ A register of people with access to provincial health insurance, which includes BC residents (Canadian citizens, permanent residents, people with visas longer than 6 months, and dependents of people in these categories residing in BC).

**BC Emergency Health Services (BCEHS):** Information about the time, nature, and location of overdose events as coded by ambulance paramedics.

**Drug and Poison Information Centre (DPIC):** Records from calls to the provincial poison hotline for poisoning management.

**BC Coroner’s Service (BCCS):** Details on all accidental and undetermined illicit drug toxicity deaths in BC.

**BC Vital Statistics:** Demographic and medical information on deaths.

**Enhance Emergency Department Records:** Additional information on opioid-related drug overdose cases treatment in emergency departments in three of five BC Health Authorities.

**National Ambulatory Care Reporting System (NACRS):**^2^ Information on ambulatory care in BC, covering 67% of ED visits in the province.

**Discharge Abstract Database (DAD):**^3^ Records of stays in acute care hospitals in BC.

**Medical Services Plan (MSP):**^4^ Billing records for all fee-for-service provider visits covered by BC’s health insurance program. Captures all primary care physician visits.

**BC Corrections:** Records of admissions and releases from BC provincial correctional centres for all people aged 18 or older.

**Social assistance:** Records of social assistance payments provided by the Ministry of Social Development and Poverty Reduction. Includes unemployment, disability, food insecurity, and housing instability.

**PharmaNet:**^5^ Record of all community pharmacy dispensations in BC.

**Dataset References**

1. British Columbia Ministry of Health [creator] (2021): Client Roster. British Columbia Ministry of Health [publisher]. Data Extract. MOH (2021).

2. Canadian Institute of Health Information [creator] (2021): National Ambulatory Care Reporting System (NACRS). British Columbia Ministry of Health [publisher]. Data Extract. MOH (2021).

3. Canadian Institute of Health Information [creator] (2021): Discharge Abstract Database (Hospital Separations). British Columbia Ministry of Health [publisher]. Data Extract. MOH (2021).

4. British Columbia Ministry of Health [creator] (2021): Medical Services Plan (MSP) Payment Information File. British Columbia Ministry of Health [publisher]. Data Extract. MOH (2021).

5. British Columbia Ministry of Health [creator] (2021): PharmaNet. British Columbia Ministry of Health [publisher]. Data Extract. MOH (2021).

## References

1. Slaunwhite A, Min JE, Palis H, Urbanoski K, Pauly B, Barker B, et al. Effect of Risk Mitigation Guidance for opioid and stimulant dispensations on mortality and acute care visits during dual public health emergencies: retrospective cohort study. BMJ. 2024 Jan 10;e076336.

2. Palis H, Zhao B, Young P, Korchinski M, Greiner L, Nicholls T, et al. Stimulant use disorder diagnosis and opioid agonist treatment dispensation following release from prison: a cohort study. Subst Abuse Treat Prev Policy. 2022 Nov 24;17(1):77.

3. B.C. Health System Strategy: Geographic Service Areas. BC Ministry of Health; 2016 Apr.
